# BODY TYPES AND ASSOCIATION WITH DEPRESSIVE SYMPTOMS AMONG OLDER ADULTS: FINDINGS FROM NHANES 2013-2016

**DOI:** 10.1093/geroni/igz038.1004

**Published:** 2019-11-08

**Authors:** Queendaleen C Chukwurah, Lydia Bazzano

**Affiliations:** 1 Tulane Center for Aging, School of Medicine, Tulane University, New Orleans, Louisiana, United States; 2 Department of Epidemiology, School of Public Health and Tropical Medicine, Tulane University, New Orleans, Louisiana, United States

## Abstract

Older Americans are increasingly affected by overweight and obesity now as compared to previous decades. We examine the prevalence rates and association of Depressive Symptoms (DS) across body types created using the National Heart, Lung, Blood Institute recommended body mass index /waist circumference (WC) anthropometric cut off values among older Americans. 3,132 participants, 50 years and older from the National Health and Nutrition Examination Survey (NHANES) 2013-2016 was used for this analysis. Six body types were created using the anthropometric cut off values- normal weight with normal WC, overweight with normal WC, obese with normal WC, normal weight with high WC, overweight with high WC, and obese with high WC. The PHQ-9 score was used to create DS categories (1-4, 5-9, 10-14, 15-19, ≥20). The relationship of body types to DS categories was assessed using weighted multinomial logistic regression. The mean (SD) sample age was 63.4 (9.2). Approximately 12.9% of participants had a PHQ-9 score of at least 10. After adjusting for age, gender, race/ethnicity and poverty-income ratio, overweight with high WC (OR 7.61, 95% CI 2.37-24.48) had high odds of moderately severe DS. Obese with high WC had high odds of mild DS (OR 1.76, 95% CI 1.22-2.52), moderate DS (OR 2.14, 95% CI 1.09-4.20) and moderately severe DS (OR 5.59, 95% CI 2.75-11.39) compared to normal weight with normal WC. We demonstrate an association of body types with DS in an aging American population and these findings would not be identified if anthropometric measures were examined separately.

